# Improvement of identification methods for honeybee specific Lactic Acid Bacteria; future approaches

**DOI:** 10.1371/journal.pone.0174614

**Published:** 2017-03-27

**Authors:** Sepideh Lamei, Yue O. O. Hu, Tobias C. Olofsson, Anders F. Andersson, Eva Forsgren, Alejandra Vásquez

**Affiliations:** 1 Department of Laboratory Medicine Lund, Section of Medical Microbiology, Lund University, Medicon Village, Lund, Sweden; 2 Department of Ecology, Swedish University of Agricultural Sciences, Uppsala, Sweden; 3 KTH Royal Institute of Technology, Science for Life Laboratory, School of Biotechnology, Division of Gene Technology, Stockholm, Sweden; Agricultural University of Athens, GREECE

## Abstract

Honeybees face many parasites and pathogens and consequently rely on a diverse set of individual and group-level defenses to prevent disease. The crop microbiota of *Apis mellifera*, composed of 13 Lactic Acid Bacterial (LAB) species within the genera *Lactobacillus* and *Bifidobacterium*, form a beneficial symbiotic relationship with each other and the honeybee to protect their niche and their host. Possibly playing a vital role in honeybee health, it is important that these honeybee specific Lactic Acid Bacterial (hbs-LAB) symbionts can be correctly identified, isolated and cultured, to further investigate their health promoting properties. We have previously reported successful identification to the strain level by culture-dependent methods and we recently sequenced and annotated the genomes of the 13 hbs-LAB. However, the hitherto applied techniques are unfortunately very time consuming, expensive and not ideal when analyzing a vast quantity of samples. In addition, other researchers have constantly failed to identify the 13 hbs-LAB from honeybee samples by using inadequate media and/or molecular techniques based on 16S rRNA gene sequencing with insufficient discriminatory power. The aim of this study was to develop better and more suitable methods for the identification and cultivation of hbs-LAB. We compared currently used bacterial cultivation media and could for the first time demonstrate a significant variation in the hbs-LAB basic requirements for optimal growth. We also present a new bacterial identification approach based on amplicon sequencing of a region of the 16S rRNA gene using the Illumina platform and an error correction software that can be used to successfully differentiate and rapidly identify the 13 hbs-LAB to the strain level.

## Introduction

Pollinators are vital for the maintenance of wild ecosystems and agricultural production. The honeybee, *Apis mellifera*, is one of the most economically important pollinators [1], also valued for its honey and wax production. Due to the loss of wild pollinators and an increasing demand for pollinators in food production, healthy honeybees are of great standing. Infectious diseases caused by pathogenic bacteria, fungi and viruses are some of the major threats to honeybees [2]. However, far from all microorganisms are harmful, and members within the phenotypically defined group called Lactic Acid Bacteria (LAB) [3–5] are generally considered beneficial microorganisms. Beneficial microorganisms such as honeybee specific Lactic Acid Bacteria (hbs-LAB) originating from the honey crop [6] have been identified in honeybees. The hbs-LAB protect honeybees from pathogens and nectar spoilers [7,8]. Bifidobacteria are included in the LAB definition due to their similar origin, their production of organic acids and common use by the food and biotech industries [9]. LAB associated with honeybees such as *Lactobacillus johnsonii* [4,5], *Lactobacillus plantarum*, *Lactobacillus brevis* [10], *Lactobacillus apis* [11] and other hbs-LAB [7,8] have been proposed to have an inhibitory effect on honeybee pathogens such as *Paenibacillus larvae* and *Melissococcus plutonius*. In addition, Olofsson and Vásquez [6] observed a decrease in the quantity of hbs-LAB when honeybees became infected with *P*. *larvae*, and it has also been shown that the concentration of *Lactobacillus* spp. decrease in diseased compared to healthy honeybee colonies [12] suggesting a significant role in honeybee health.

In this study we focused on a hbs-LAB microbiota composed of nine *Lactobacillus* and four *Bifidobacterium* species constantly found in the honeybee crop and their food products [6,13] and consistent regardless of the geographic origin of the honeybees [8,14,15]. This microbiota has been evolutionarily shaped to defend honeybees and their habitat from incoming microbial threats by producing different metabolites and proteins with potential antimicrobial activity [8,16,17]. Hbs-LAB colonize the honey crop despite its harsh conditions such as a constant nectar flow, high osmotic pressure and a constant inflow of microorganisms from the environment. They also end up in bee bread, bee pollen [8,18] and fresh honey [8] as they play a role in the fermentation of pollen and production of honey.

Nectar and pollen are the main food sources for honeybees, providing carbohydrates (such as glucose, fructose and sucrose) [19], minerals, lipids, vitamins, protein and amino acids such as L-cysteine [20]. Since the hbs-LAB colonize the honey crop where carbohydrates are abundant, it is not surprising that they require supplements such as fructose and L-cysteine rather than glucose to grow under laboratory conditions. The growth media initially used to culture the hbs-LAB [6] was later replaced by MRS supplemented with fructose and L-cysteine (sMRS), to better mimic the hbs-LABs natural environment and growth conditions [7,8,13,16,17,21–23]. However, these vital supplements (fructose and L-cysteine) have not been used in bacterial cultivation by other researchers [24–26], possibly explaining the inability to isolate more than a few hbs-LAB.

LAB species occurring in low numbers are often out-competed *in vitro* by numerically more abundant microbial species making isolation of the less abundant bacterial strains difficult. As a result, ecological studies increasingly rely on culture-independent methods based on DNA or RNA analyses [27]. The hitherto applied molecular approaches used in various studies have failed to identify and differentiate all individual species and strains of the hbs-LAB microbiota originating from the honey crop [24–26,28]. Inadequate isolation of genomic bacterial DNA, a key step in molecular biology techniques, may be another explanation for underestimating the number of hbs-LAB strains present in honeybees. Like many other LAB such as *Streptococcus thermophiles* [29], the hbs-LAB are very resistant and weakly susceptible to lysozyme. One should consider that their robust cell wall structure may demand for particularly harsh DNA extraction protocols.

The Illumina platform has with its advantages (high-throughput capacity, high-accuracy and cost-effectiveness), has been considered the gold standard in microbial diversity analysis [30,31]. Until recently, a significant challenge of Illumina rRNA gene amplicon sequencing has been to accurately identify true biological sequence variants while excluding amplicon sequencing errors [32–34]. A common way to study microbial communities is to cluster similar amplicon reads, which masks most amplification errors [33,35,36], but at the same time removes some true biological variants. It has been shown that the hbs-LABs 16S rRNA genes have highly similar sequences [22,23] although they represent different species. This may explain why there are few reports on hbs-LAB using Illumina sequencing of the 16S rRNA genes [24–26,28]. Due to the limitations of culture-dependent as well as molecular techniques, a combination of both is recommended for future accurate investigations of the hbs-LAB.

Considering the presumptive important role that hbs-LAB play in honeybee health, using adequate techniques to identify, differentiate and isolate them are of key importance for this research area. Consequently, the aim of the present study was to demonstrate the requirements for vital growth supplements of the 13 hbs-LAB strains by monitoring growth rates. Another objective was to evaluate a novel identification approach to differentiate the hbs-LAB strains using a recently published Illumina amplicon error-correction program [32] for retrieving 16S rRNA gene sequences from Illumina sequences.

## Methods

### Honeybee specific LAB and culture conditions

Thirteen hbs-LAB strains, nine Lactobacilli (*L*. *kunkeei* Fhon2N, *L*. *apinorum* Fhon13N, *L*. *mellis* Hon2N, *L*. *mellifer* Bin4N, *L*. *apis* Hma11N, *L*. *helsingborgensis* Bma5N, *L*. *melliventris* Hma8N, *L*. *kimbladii* Hma2N, *L*. *kullabergensis* Biut2N) and four Bifidobacteria (*B*. *asteroides* Bin2N, *B*. *asteroides* Bin7N, *B*. *asteroides* Hma3N and *B*. *coryneforme* Bma6N), were originally isolated and identified as previously described [6,8,13]. All strains were incubated individually and anaerobically at 35°C in non-supplemented Man, Rogosa & Sharpe (nsMRS) (OXOID LTD, England) broth and in MRS broth supplemented with 2% fructose (Merck, Sollentuna, Sweden) and 0.1% L-cysteine (Sigma-Aldrich, Stockholm, Sweden), (sMRS).

### Growth curves

Non-supplemented MRS and supplemented MRS broth were inoculated with each hbs-LAB strain individually and their growth monitored every 2 hours (h) for 3 days of incubation by measuring the optical density (OD) at λ = 600 nm in a spectrophotometer (Gynesys, ThermoSpectronic, USA). Uninoculated nsMRS and sMRS were used as background controls. The analyses were carried out in triplicates and the mean calculated and presented in the graphs. All statistical analyses of growth curves were performed using the software package GraphPad Prism 6.00 for Windows (GraphPad Software, San Diego, CA, USA).

### DNA extraction

DNA was extracted using DNeasy Blood and Tissue kit (Qiagen, Germany) according to the manufacturer’s protocol with minor modifications. Briefly, ten bacterial colonies were transferred to Eppendorf tubes containing 30 μl of sterile water. The tubes were vortexed and incubated at 70°C for 30 minutes. After cooling at room temperature, 10 μl of human lysozyme (70 000 units; Sigma, Aldrich, Germany) and 180 μl of lysis buffer [100 mM Tris-HCl pH = 8.0, 2 mM EDTA, 0.05% Triton X (Sigma, Aldrich, USA)] were added and the samples were incubated on a shaker at 37°C for 18 h. DNA purity and quantity was determined using a Nano-Drop (ND 8000 UV/Vis Spectrophotometer, Thermo Fisher Scientific, USA). Extracted DNA was stored at -20°C until further analysis.

### PCR and sequencing

In order to enable sequencing from multiple samples in one go, we applied a procedure with three consecutive PCRs for the amplicon library. The first PCR procedure targeting the V1-V2 regions (position 8–357 *E*. *coli* numbering, fragment length of ~ 320 bp) of the 16S rRNA gene, with a set of primers ENV1 (5′-AGAGTTTGATXXTGGCTCAG-3′, X = Inosine) and TGEE7 (5′-CTGCTGCCTCCCGTAGG-3′) [37] used for the next PCR steps after adding adapters to the amplicons. The sequences of all primers and the reaction mixture content of each PCR procedure are listed in S1 File. Equimolar amounts of indexed amplicon samples were pooled for the amplicon library and sequenced together with 10% of PhiX in one lane of an Illumina MiSeq (MiSeq Control Software 2.4.1.3 and RTA 1.18.54) using MiSeq Reagent Kit v3 (600 cycles, read lengths are 2x300bp). The 13 hbs-LAB strains were sequenced in two MiSeq runs (DNA material extracted from the strains Bin2N and Hma8N were pooled in one sequencing run and the others were pooled in another run).

### Sequence processing

Since each bacterial strain was expected to contain only a few variants of 16S rRNA gene sequences [38] we subsampled 10,000 read pairs from each strain for the data analysis (except Hma8N, which contained only 6,664 reads and for which we used all reads). Since the DADA2 program retrieves biological sequences from reads by modeling the Illumina-sequencing errors [32], and different sequencing runs might have distinct qualities, we ran the DADA2 pipeline (v.1.1.1, http://benjjneb.github.io/dada2/tutorial.html) on our samples from the two runs separately. After examining the quality profiles of forward and reverse reads, we trimmed off the low-quality tails from the 3’ ends to make the filtered reads having an average Phred score Q >30 (the lengths of truncated forward and reverse reads were 230 and 150 bp, respectively), meanwhile, we required no N (maxN = 0), no PhiX sequences (rm.phix = TRUE) and the maximum number of expected error being 2 (maxEE = 2) for all paired-end reads. With the summary of the quality information for each unique sequence, the core denoising algorithm of DADA2 was performed on the forward and reverse reads separately to infer the real sample sequences, in which the error rates and sample sequences were alternately estimated until convergence. In the merging step of the paired reads, >30 bp overlap was required with no mismatches. After checking chimeric sequences from the denoised forward and reverse sequences (function “isBimeraDenovo”) and removing them from the merged sequences, a total of 22 sequence types were finally inferred from the 13 hbs-LAB strains. To evaluate whether the DADA2 inferred 16S rRNA sequences corresponded to the 13 hbs-LAB strains’ genetic content, the obtained sequences were BLAST-searched against the NCBI nt database for taxonomic information. They were also compared to the 16S rRNA gene sequences (Sanger sequenced reference sequences) of the 13 hbs-LAB strains for examining the differences between the inferred sequences and the reference sequences. A phylogenetic tree was built with FastTree (v2.1.8) [39] after aligning all sequences with Clustal Omega [40]. FigTree (v1.4.2, http://tree.bio.ed.ac.uk/software/figtree/) was used for tree visualization.

## Results

### Growth of honeybee specific LAB

**Fig 1:**
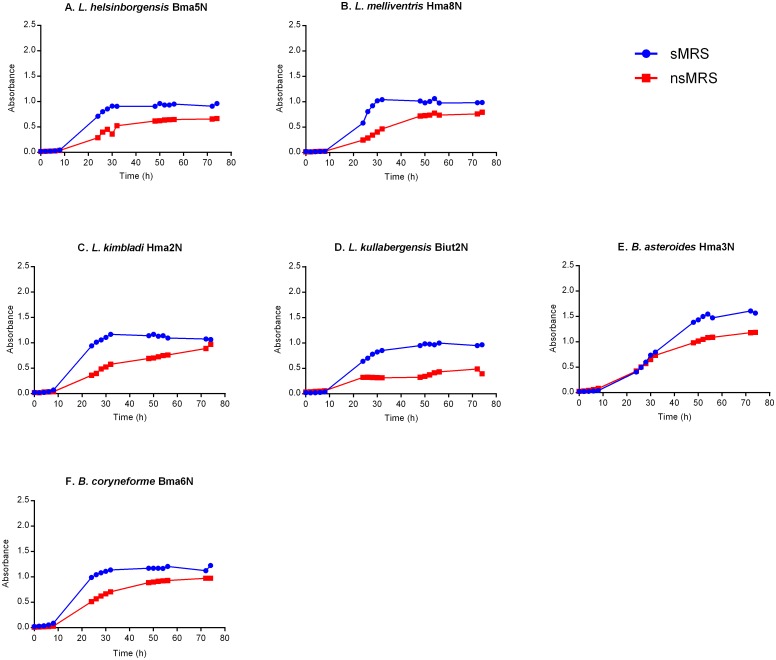
Growth curves of hbs-LAB in non-supplemented MRS (red) and supplemented-MRS (blue) at measured OD (λ 600 nm).

Only six of the 13 hbs-LAB strains, four Lactobacilli (Bma5N, Hma8N, Hma2N and Biut2N) and two Bifidobacteria (Hma3N and Bma6N), were able to grow in nsMRS. Their growth was significantly better and increased about two-fold in supplemented media (Fig 1). For instance, strains Biut2N and Hma2N doubled their cell density in sMRS (0.85 and 1.16 at λ = 600 nm) compared to the growth in nsMRS (a cell density of 0.31 and 0.58 respectively after 32 h of incubation).

**Fig 2:**
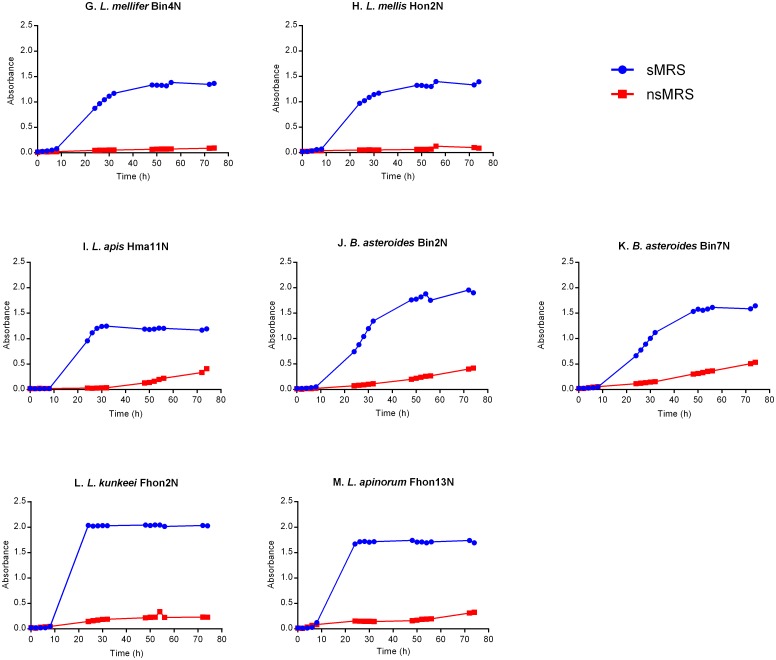
Growth curves of hbs-LAB in non-supplemented MRS (red) with supplemented-MRS (blue) at measured OD (λ 600 nm).

The remaining seven strains, including five Lactobacilli (Hon2N, Bin4N, Hma11N, Fhon2N and Fhon13N) and two Bifidobacteria (Bin2N and Bin7N), were only able to grow in sMRS medium (Fig 2).

As previously shown [17], the bacterial strains Fhon2N and Fhon13N, grew faster than the other hbs-LAB in sMRS and reached early stationary phase after 24 h. Fhon2N reached the highest cell density (Fig 2) at early stationary phase, followed by Bin2N and Fhon13N (Fig 2). Biut2N and Bma5N strains (Fig 1) had the lowest cell density at early stationary phase in sMRS. Some strains such as Bin2N, Bin7N and Hma3N, grew slower and reached early stationary phase after around 54 h (Figs 1 and 2). The remaining strains (Hon2N, Bin4N, Hma11N, Bma5N, Hma2N, Hma8N, Biut2N and Bma6N) reached early stationary phase after approximately 30–32 h incubation in sMRS (Figs 1 and 2).

### 16S rRNA sequencing of hbs-LAB

**Fig 3:**
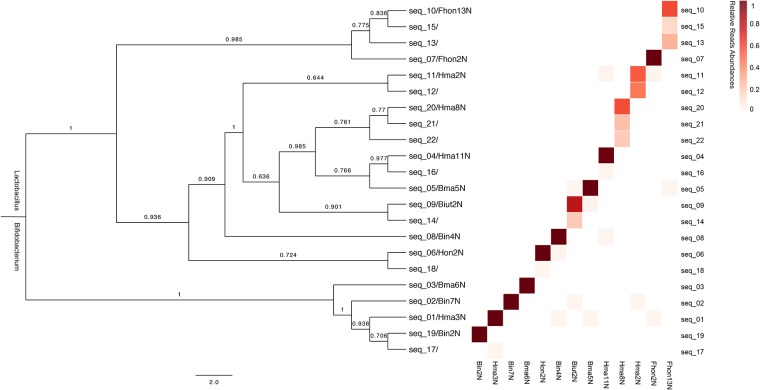
Phylogenetic tree and abundance distribution of hbs-LAB 16S rRNA sequences inferred in this study. The dendrogram illustrates the phylogenetic relationship of the 22 sequence types. Leaf labels marked with hbs-LAB strain names indicate sequences identical to the reference 16S rRNA sequence. The relative abundance of sequence types for each strain is shown in the heat map (each column represents one strain).

The V1-V2 regions of the 16S rRNA genes of each of the 13 hbs-LAB strains were sequenced. A subsample of 10,000 read pairs was analyzed for each strain (except for Hma8N, where only 6,664 reads existed). After error correction and removing chimeras using the DADA2 software, 22 sequence types remained representing 80.2% of the total reads. These 22 sequence reads were all classified to the genus level as *Lactobacillus* or *Bifidobacterium* by online BLAST searches against the NCBI nt database. BLAST searches against reference sequences of hbs-LAB strains showed that the most abundant sequence type of each strain was 100% identical to the corresponding reference sequence. Nine of the 13 strains were dominated (> 99.6% of reads) by a single sequence type while the remaining strains where dominated by two or three sequence types. In order to inspect the relationship of the hbs-LAB sequences, we built a phylogenetic tree on the 22 sequences (Fig 3). For strains Biut2N and Hma2N that were dominated by two sequence types, the less abundant sequence had a one base substitution compared to the most abundant sequence (or the reference sequence). For strains Fhon13N and Hma8N, with three abundant sequence types, had two variant sequences with maximum two bases difference to their dominant sequence. The sequencing reads have been submitted to the European Nucleotide Archive (ENA) under accession number PRJEB18095.

## Discussion

We compared growth media traditionally used for isolation of hbs-LAB and show that the investigated supplements are essential for their growth. Our data also confirm that the addition of L-cysteine and fructose is crucial for the optimal growth of hbs-LAB under laboratory conditions [16,17,21]. Their need for such substances is not surprising since their natural habitats, honey crops, honey [6,8] and bee bread, contain foraged nectar and pollen [18] rich in amino acids and sugars. Nectar consists mainly of sucrose and its monosaccharides fructose and glucose, but the exact composition of sugars differs between continents, seasons and floral sources [19]. Consequently, hbs-LAB had to adapt to carbohydrate-rich environment. For instance, during specific habitat adaptions, LAB may acquire or lose genes reflecting a shift toward a nutrient-rich lifestyle [41]. Reductive genome evolution has been reported for obligate host-associated pathogens and symbionts, where gene loss and degradation correlate with the shift to the intracellular lifestyles [42]. A recent genomic study of hbs-LAB by Ellegaard and colleagues showed that hbs-LAB have many accessory genes related to carbohydrate transport and storage [22]. This was also observed in *L*. *kunkeei* and *L*. *apinorum* [23], suggesting further adaption of the hbs-LAB to their habitats. Unlike *Lactobacillus plantarum* with the largest genome size known for LAB (3.3 Mb) [43], hbs-LAB possess small genomes, ranging from 1.54 Mb (Hma11) to 2.13 Mb (Hma2) [22,23]. It has been suggested that the large size of the *L*. *plantarum* genome could be related to the diversity of the environmental habitats of this bacterium, while hbs-LAB have evolved in a nutritionally rich environment [22,23]. The characteristic of a small genome and the shift to a nutrient-rich lifestyle has been reported also in *Fructobacillus* species [44]. Moreover, there is a duplication of genes involved in transport and metabolism of carbohydrates enhancing the ability of LAB [41] and hbs-LAB [22] to exploit sugar-rich environments.

It has been shown that the presence of varying carbohydrate sources (*e*.*g*. hexoses, pentoses, sugar alcohols) and other supplements have effect on bacterial growth and metabolic pathways [45,46]. For example, *Lactobacillus helveticus* require amino acids, vitamins and bases for the growth in a glucose-salt defined medium [47]. Killer *et al*. used a very rich medium (wheat germ medium), containing free amino acids and peptides to isolate *L*. *apis*, one of the 13 hbs-LAB members [11]. This is in line with the results from our study showing that the hbs-LABs require media supplemented with fructose and L-cysteine to grow (Fig 2) and that bacterial growth increases about two-fold in sMRS (Fig 1).

Interestingly, *L*. *kunkeei* has been classified as an obligate fructophilic bacteria preferring fructose as sugar source and being able to grow well on glucose only in the presence of fructose and external electron acceptors [48,49,50]. Later, it was found that *L*. *kunkeei* is one of the 13 hbs-LAB strains not only present in the honey crop [8,27], but also abundant in beebread [18,25], pollen [18], honeybee larvae [51] as well as in wines [52], flowers and fruits which are rich in fructose [25,48,49]. Noticeably, the growth media traditionally used to isolate honeybee hbs-LAB microbiota in other studies [24–26] were not supplemented, which explain why no findings of the 13 hbs-LAB strains were reported.

A high similarity of 16S rRNA gene sequences has been documented in hbs-LAB strains [22] and other honeybee gut bacteria such as *Gilliamella apicola*, *Snodgrasella alvi* [53] and two *Lactobacillus* spp. strains, wkB8 and wkB10 [54]. Such high sequence similarity makes it difficult to differentiate bacteria to the strain level using 16S rRNA amplicon data usually targeting short gene regions and entailing errors introduced during the PCR and sequencing. Moran *et al*. recently showed that deep sequencing of short amplicons of 16S rRNA genes (V4 region) is insufficient to identify *Snodgrassella alvi* at the strain level [22]. However, in this study, we successfully retrieved the 16S rRNA gene sequences for the 13 hbs-LAB strains using Illumina amplicon sequencing and the DADA2 software for error correction. For four of the Lactobacilli strains (Biut2N, Hma2N, Hma8N, Fhon13N), multiple but highly similar sequence types were identified. The most likely reason for this is intra-genomic heterogeneity of 16S rRNA genes, a common phenomenon when species hold multiple copies of the 16S rRNA gene [55,56]. In a previous study, the 13 hbs-LAB strains were confirmed to have multiple copies of the 16S rRNA gene [22]. In *Escherichia coli*, the use of multiple rRNA operons has been shown to facilitate shifts from poor to rich growth conditions [57]. Thus, possessing multiple copies of the 16S rRNA gene could indicate that hbs-LAB strains may have evolved under selection for rapid growth following shifts from poor to nutritionally rich environments. Besides the major sequences, some strains had a few sequence reads that matched dominant sequences of other strains, possibly caused by cross-contamination when preparing the sequencing library.

Due to biases and limitations of the commonly used techniques discussed earlier, we suggest a simple approach for accurate identification of the 16S rRNA sequence variants of the hbs-LAB symbionts to strain level by combining targeted sequencing of the V1-V2 regions of the 16S rRNA gene and the DADA2 software for analysis of Illumina data. However, a true understanding of the physiology of the hbs-LAB and the role they play in their ecological niches and in honeybee health is based on a deeper understanding of mechanisms of their actions, requiring optimal bacterial cultivation with supplemented media. As a result of the limitations in current culture-dependent and molecular techniques, we recommend using a combination of both. They are suitable as complementary techniques for future investigations of the hbs-LAB and other members of the honeybee bacterial gut microbiome.

## Conclusion

We demonstrated, for the first time, that it is imperative to use supplements such as fructose and L-cysteine to successfully isolate and grow hbs-LAB. In earlier studies using conventional media (non-supplemented MRS), most hbs-LAB have possibly been misidentified or failed to be isolated. This also ties in with unsuccessful identification of hbs-LAB to strain level or misidentifying certain similar strains. To our knowledge, this is the first report that successfully identifies the 13 hbs-LAB to the strain level using a novel approach based on Illumina amplicon sequencing of the 16S rRNA genes. The improved methods presented here are reliable techniques for isolation, identification and differentiation of hbs-LAB to the strain level and show great promise to be considered as future standards for studies of the honeybee LAB microbiota.

## Supporting information

S1 File PCR procedure and Illumina index primers of the 13 hbs-LAB. (DOCX)
