# Supplementary material for: Improvement of identification methods for honeybee specific Lactic Acid Bacteria; future approaches
Source: PLoS One. 2017 Mar 27;12(3):e0174614. doi: 10.1371/journal.pone.0174614 (PMC5367889; doi:10.1371/journal.pone.0174614)
Supplement: S1 File — (DOCX) [file pone.0174614.s001.docx]

# S1 File

A three-step PCR were conducted to amplify V1-V2 region from the 16S rRNA gene of the 13 LAB strains and to barcode these amplicons to allow them to be sequenced in one lane. The information about the PCR primer, mixture content and condition were described in the following. Between each PCR step, the amplicon products were cleaned using a Qiagen extract gel kit (QIAquick, Qiagen, Germany) to eliminate short DNA fragments (e.g. loose primers, primer dimers).

## The first PCR step

Attached ENV1 and TGEE7 primers to Illumina adapters (S Table 1) were used to amplify V1-V2 regions of 16S rRNA gene and, meanwhile, to attach the adapter sequences for the next PCR step. The amplicon products were used in the next step. The reaction mixture (50 µl) contained 0.5 µM of each primer, 0.2 mM of each deoxyribonucleotide triphosphate (Thermo Fisher Scientific, USA), 1× Phusion HF buffer, 0.02 U/μl Phusion DNA polymerase (Thermo Fisher Scientific) in all steps. After an initial denaturation step at 95 °C for 2 min, 30 cycles of denaturation at 96 °C for 15 seconds, annealing at 48°C for 30 sec and extension at 72 °C for 1 min and 30 sec were performed, followed by a final extension step at 72 °C for 10 min.

**Primer sequences of the first PCR step**

| **Primer name** | **Primer structure** | **Primer sequence** |
| --- | --- | --- |
| **Forward_primer _1** | Illumina adapter-ENV1 | ACACTCTTTCCCTACACGACGCTCTTCCGATCT-AGAGTTTGATXXTGGCTCAG |
| **Reverse_primer _1** | Illumina adapter-TGGE7 | AGACGTGTGCTCTTCCGATCT-CTGCTGCCTCCCGTAGG |

## The second PCR step

This PCR step is to extend the length of the 16S amplicons to enable them to be sequenced and indexed. The sequence that can align to Illumina sequencing flowcell was added to the 5’-end of the 16S amplicons and the sequence that can align to the indexing primers was added to the 3’-end.

The PCR template of this step is the cleaned amplicon yielded in the first PCR. The PCR reaction and program is the same as the previous step.

**Primer sequences of the second PCR step**

| **Primer name** | **Primer sequence** |
| --- | --- |
| **Forward_primer _2** | AATGATACGGCGACCACCGAGATCTACACTCTTTCCCTACACGACGCTCTTCCGATCT |
| **Reverse_primer _2** | GTGACTGGAGTTCAGACGTGTGCTCTTCCGATCT |

## The third PCR step

In this step, 13 indexing primers that contain distinct 6-base barcode and the sequence that can align to the Illumina flowcell surface were applied to the 16S amplicons of each LAB strain. These indexing primers align to the 3’-end of the second PCR yielded amplicons, which enable us to pool all amplicons together and sequence the library in one lane. The PCR reaction was prepared as mentioned in previous steps. The annealing temperature of this step was increased to 52°C.

**Reverse primer sequences of the third PCR step**

| **Primer name** | **Primer sequence** |
| --- | --- |
| **Primer_Hon2N** | CAAGCAGAAGACGGCATACGAGAT-**ATCACG**-GTGACTGGAGTTC |
| **Primer_Fhon2N** | CAAGCAGAAGACGGCATACGAGAT-**CGATGT**-GTGACTGGAGTTC |
| **Primer_Fhon13N** | CAAGCAGAAGACGGCATACGAGAT-**TTAGGC**-GTGACTGGAGTTC |
| **Primer_Hma11N** | CAAGCAGAAGACGGCATACGAGAT-**TGACCA**-GTGACTGGAGTTC |
| **Primer_Biut2N** | CAAGCAGAAGACGGCATACGAGAT-**ACAGTG**-GTGACTGGAGTTC |
| **Primer_Hma2N** | CAAGCAGAAGACGGCATACGAGAT-**GCCAAT**-GTGACTGGAGTTC |
| **Primer_Bma5N** | CAAGCAGAAGACGGCATACGAGAT-**CAGATC**-GTGACTGGAGTTC |
| **Primer_Hma8N** | CAAGCAGAAGACGGCATACGAGAT-**ACTTGA**-GTGACTGGAGTTC |
| **Primer_Bin4N** | CAAGCAGAAGACGGCATACGAGAT-**GATCAG**-GTGACTGGAGTTC |
| **Primer_Bma6N** | CAAGCAGAAGACGGCATACGAGAT-**TAGCTT**-GTGACTGGAGTTC |
| **Primer_Hma3** | CAAGCAGAAGACGGCATACGAGAT-**GGCTAC**-GTGACTGGAGTTC |
| **Primer_Bin2** | CAAGCAGAAGACGGCATACGAGAT-**CTTGTA**-GTGACTGGAGTTC |
| **Primer_Bin7** | CAAGCAGAAGACGGCATACGAGAT-**AGTCAA**-GTGACTGGAGTTC |
